# Plantar pressure distribution during gait and running in subjects with chronic ankle instability

**DOI:** 10.1186/1757-1146-5-S1-P32

**Published:** 2012-04-10

**Authors:** Roel De Ridder, Tine Willems, Philip Roosen

**Affiliations:** 1 Departement of Rehabilitation Sciences and Physiotherapy, Ghent University, Ghent, 9000, Belgium

## Background

**Figure 1:**
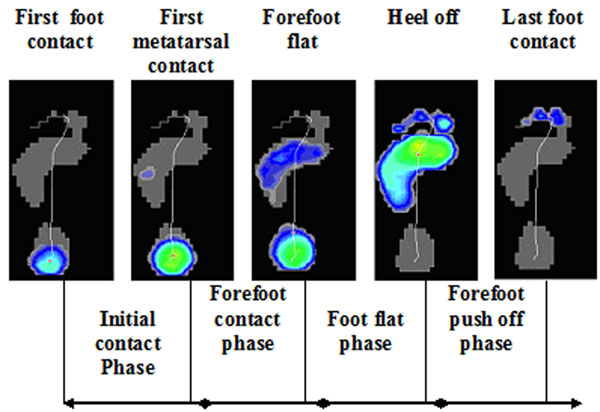
Five distinct moments and phases relative to total foot contact [2]

Lateral ankle sprains are one of the most common injuries in athletes. Up to 32% of subjects with an ankle sprain develop residual symptoms labeled as chronic ankle instability (CAI), with a significant impact on the quality of life. In spite of many research the underlying mechanisms for CAI remain unclear. The foot roll-off pattern of subjects with CAI is one of the factors which may play an important role in recurring ankle sprains and the presence of ‘giving way’ episodes. A more lateral pressure distribution has been suggested in subject with CAI, resulting in higher risk for developing an ankle sprain, but research is limited [1]. Especially in dynamic conditions research is needed. This study includes gait as well as a running condition and investigates plantar pressure distribution on five distinct moments and during 4 phases relative to total foot contact as shown in figure 1 [2].

## Materials and methods

Plantar pressure distribution of 93 subjects (42 subjects with CAI, 21 copers and 29 healthy subjects) was registered during barefoot walking and running. Data was collected on a 20m long runway with a Footscan® pressure plate imbedded on top of a forceplate (AMTI). Medio-lateral ratios were calculated for the five distinct moments and during the four phases of the stance phase. Temporal data, peak pressure, mean force and impulse for the different zones of the foot were also calculated.

## Results

Statistical analysis did not show significant differences between the groups for any of the tested parameters nor for the medio-lateral ratios at the different moments and during the phases.

## Conclusions

This study does not confirm the results of previous studies suggesting a more lateral pressure distribution. Further research is needed.
